# Phytohormone Signaling of the Resistance to *Plum pox virus* (PPV, Sharka Disease) Induced by Almond (*Prunus dulcis* (Miller) Webb) Grafting to Peach (*P. persica* L. Batsch)

**DOI:** 10.3390/v10050238

**Published:** 2018-05-03

**Authors:** Azam Nikbakht Dehkordi, Manuel Rubio, Nadali Babaeian, Alfonso Albacete, Pedro Martínez-Gómez

**Affiliations:** 1 Faculty of Crop Science, Department of Plant Breeding and Biotechnology, Sari Agricultural Sciences and Natural Resources University (SARNU), Km 9, Darya Road P.O. Box 578 Sari, Iran; azam.nikbakht.dehkordi@gmail.com (A.N.D.); nbabaeian@yahoo.com (N.B.); 2 Department of Plant Breeding, Centro de Edafología y Biología Aplicada del Segura-Consejo Superior de Investigaciones Científicas (CEBAS-CSIC), P.O. Box 164, 30100 Espinardo, Murcia, Spain; mrubio@cebas.csic.es; 3 Department of Plant Nutrition, Centro de Edafología y Biología Aplicada del Segura-Consejo Superior de Investigaciones Científicas (CEBAS-CSIC), P.O. Box 164, 30100 Espinardo, Murcia, Spain; alfmoreno@cebas.csic.es

**Keywords:** *Prunus*, virus resistance, PPV, sharka, phytohormones, breeding

## Abstract

*Plum pox virus* (PPV, sharka) is a limiting factor for peach production, and no natural sources of resistance have been described. Recent studies, however, have demonstrated that grafting the almond cultivar “Garrigues” onto the “GF305” peach infected with Dideron-type (PPV-D) isolates progressively reduces disease symptoms and virus accumulation. Furthermore, grafting “Garrigues” onto “GF305” prior to PPV-D inoculation has been found to completely prevent virus infection, showing that resistance is constitutive and not induced by the virus. To unravel the phytohormone signaling of this mechanism, we analyzed the following phytohormones belonging to the principal hormone classes: the growth-related phytohormones cytokinin trans-zeatin (tZ) and the gibberellins GA_3_ and GA_4_; and the stress-related phytohormones ethylene acid precursor 1-aminocyclopropane-1-carboxylic acid (ACC), abscisic acid (ABA), salicylic acid (SA), and jasmonic acid (JA). PPV inoculation produced a significant increase in GA_3_ and ABA in peach, and these imbalances were related to the presence of chlorosis symptoms. However, grafting “Garrigues” almond onto the PPV-inoculated “GF305” peach produced the opposite effect, reducing GA_3_ and ABA contents in parallel to the elimination of symptoms. Our results showed the significant implication of SA in this induced resistance in peach with an additional effect on tZ and JA concentrations. This SA-induced resistance based in the decrease in symptoms seems to be different from Systemic Acquired Resistance (SAR) and Induced Systemic Resistance (ISR), which are based in other reactions producing necrosis. Further studies are necessary, however, to validate these results against PPV-D isolates in the more aggressive Marcus-type (PPV-M) isolates.

## 1. Introduction

*Plum pox virus* (PPV, sharka disease) is a threat to the production of stone fruits (*Prunus*) in affected areas. PPV was first observed in plums in Bulgaria between 1915 and 1918, although some reports indicate that symptoms were seen in Macedonia as early as 1910. Since then, PPV has spread throughout Europe, the Mediterranean basin, the Middle East (Egypt and Syria), India, and South and North America [1]. In Europe alone, according to estimates from the European and Mediterranean Plant Protection Organization (EPPO), more than 100 million stone fruit trees have been infected with PPV [2]. The constant progress of sharka in Europe and the severity of the disease led to the development of the Sharka International Working Group in the 1970s within the framework of the EPPO, which made coordinated research and the free flow of information between countries possible [2]. Similar to other plant viruses, *Plum pox* comprises several strains based on biology, serological reactions, and molecular and biological data. To date, nine PPV strains have been recognized (PPV-D, PPV-M, PPV-Rec, PPV-EA, PPV-C, PPV-T, PPV-W, PPV-CR, and PPV-An). M (Marcus) is very aggressive, while D (Dideron) is a less aggressive type. Another isolate, El Amar, has also been reported, which some authors claim belongs to type M and others identify as type D. Recently, another isolate has been reported in cherry in Moldavia (PPV-SC), and has been sequenced, characterized, and classified as a new type C. These new types show the dangerous capacity of the virus to mutate and change. PPV-D and PPV-M, however, are the most widely distributed strains of plum pox worldwide. These strains are only partially differentiable by their biological or epidemiological properties. While both of these strains can infect peach, nectarine, plum, and apricot, M is much more aggressive in peach and spreads rapidly in orchards via aphids. PPV-M is also the only strain reported to transmit the virus through seed [1,3].

In the case of peach (*P. persica* (L.) Batsch), no natural sources of resistance have been identified for either strain, D or M [4,5], although M is much more aggressive in peach than D [3,5]. Nevertheless, tolerant behaviour against M isolates has recently been described in some genotypes [6], reducing sharka symptoms but not completely eliminating the virus. In addition, other studies have demonstrated that grafting the almond (*P dulcis* (Miller) Webb) cultivar “Garrigues” onto “GF305” (a very PPV-susceptible indicator) peach seedlings heavily infected with Dideron-type (PPV-D) isolates can progressively reduce disease symptoms and virus accumulation [7,8]. This response appears to be specific between almonds and peach. Furthermore, grafting “Garrigues” onto “GF305” prior to PPV-D inoculation has completely prevented virus infection, showing that resistance is constitutive and not induced by the virus [8].

Phytohormones control numerous plant physiology processes, such as germination and growth. In addition, their role in the response to biotic stress has been extensively described [9,10,11,12]. Viral infections in susceptible plants cause a hormonal disruption in which antagonistic hormones are simultaneously induced, whereas in resistant varieties, the accumulation of antagonistic hormones shows a sequential pattern [13,14]. The axis of hormonal communication within plants is polarized in two antagonistic hormonal routes: the salicylic acid (SA) and jasmonic acid (JA) route on the one hand, and the ethylene route on the other hand [15,16,17]. Other hormones are also involved in plant responses to biotic stress, such as auxins, brasinosteroids, cytokinins, and abscisic acid (ABA), which play a role in plant growth and development and are increasingly being recognized for their relevance in plant–virus interactions [18,19]. Viruses can even manipulate plant hormone responses to disarm defense mechanisms and reprogram the cellular environment to enhance replication and spread [20].

In the case of *Prunus* species, studies of hormone balance changes have focused on the following topics: physiological and growth aspects related to the response to drought resistance in plum (*P. salicina* Lindl.) [21]; the induction of multiple shoots from calli derived from stem explants [22]; the organogenesis in peach explants in in vitro multiplication conditions [23]; and the bud break in sweet cherry (*P. avium* L.) [24]. In terms of hormone signaling in the *Prunus* response to pathogens, however, only Sofo et al. [25] have analyzed the response to *Trichoderma harzianum* in cherry rootstock, but as far as we know, no hormone signaling studies have been performed on the *Prunus* response to viruses.

To unravel the nature of the induced PPV-D resistance in peach by almond grafting and its hormone signaling, different phytohormones related to growth and stress were analyzed after PPV infection in both the control “GF305” peach and that grafted with “Garrigues” almond.

## 2. Materials and Methods

### 2.1. Plant Material

**Figure 1:**
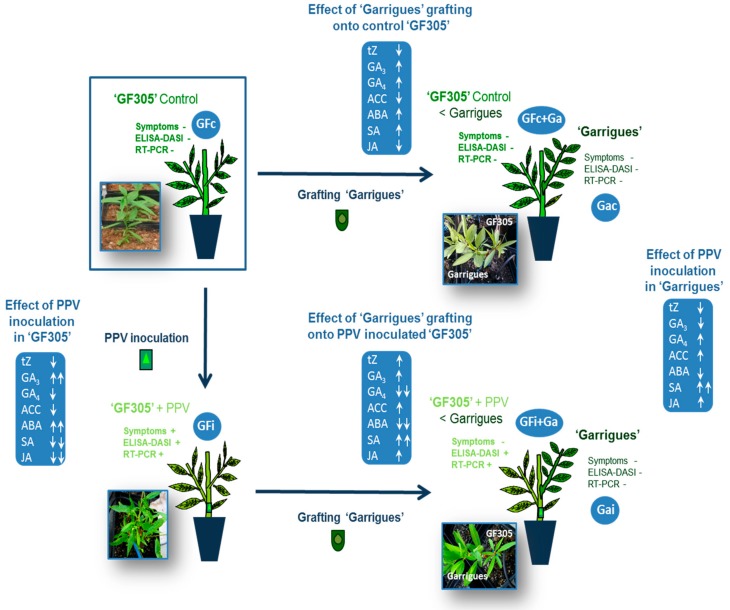
Different plant models tested: GFc = control “GF305” peach rootstock (healthy); GFi = infected “GF305” peach rootstock showing strong sharka symptoms; GFc+Ga = “GF305” peach rootstock grafted with “Garrigues” almond; GFi+Ga = “GF305” peach rootstock infected with *Plum pox virus* (PPV) without sharka symptoms after being grafted with “Garrigues” almond. Gac = Garrigues almond grafted onto healthy “GF305” rootstock; and Gai = Garrigues almond grafted onto infected “GF305” rootstock. ABA (abscisic acid); ACC (1-aminocyclopropane-1-carboxylic acid); DASI (Double antibody sandwich indirect); GA_3_ (gibberellin 3); GA_4_ (gibberellin 4); JA (jasmonic acid); SA (salicylic acid); tZ (cytokinin trans-zeatin).

“GF305” peach seedlings are characterized by their susceptibility to fruit viruses, including PPV [26]. These seedlings have been used as a rootstock in several PPV resistance tests on *Prunus* under greenhouse conditions [7,27]. In the current study, “GF305” peach was used as rootstock for grafting experiments with the “Garrigues” almond cultivar. We first analyzed control and PPV-inoculated “GF305” peach plants showing sharka symptoms. We then analyzed almond-grafted peach (inoculated and control) plants to study the phenomenon of induced resistance (Figure 1). In these grafted plants, in order to identify the mechanism of systemic defense against PPV and the effector molecule that intervenes, we conducted a differential hormone profile analysis.

### 2.2. Plum pox virus (PPV) Isolate

The PPV isolate used was 3.30RB/GF-IVIA (GenBank: KJ849228.1), a PPV-D isolate obtained from the “Red Beaut” japanese plum variety in Spain in the 1980s and maintained in the PPV collection of the Instituto Valenciano de Investigaciones Agrarias (IVIA) in Valencia (Spain). This isolate produces strong sharka symptoms in young leaves consisting of veinal chlorosis and rings (Figure 1).

### 2.3. Evaluation of Sharka Symptoms and PPV Detection

The evaluation of resistance was carried out in controlled conditions in a sealed greenhouse according to the procedure described by Rubio et al. [27]. The “GF305” peach seedlings grafted with “Garrigues” almond and the control and inoculated “GF305” seedlings were submitted to an artificial dormancy period in a cold chamber at 7 °C and darkness for about one month and then transferred to an insect-proof greenhouse for three months in the cycles of study. Eight replicates were studied for each treatment during two cycles of growth in the greenhouse. Sharka symptoms on leaves were scored using a scale of 0 (no symptoms) to 5 (maximum intensity), taking into account intensity and distribution in the plant as follows: (0) no symptoms; (1) discrete chlorosis or spots restricted to one or two leaves; (2) slight chlorosis bordering leaf veins on three or more leaves; (3) vein chlorosis or rings on numerous leaves; (4) chlorosis, rings, and some distortions on most leaves; and (5) strong chlorosis or distortions on all leaves. The presence of PPV was confirmed by ELISA-DASI (Double antibody sandwich indirect) with the specific monoclonal antibody to the capside protein (CP) of PPV 5B-IVIA/AMR (Plant Print Diagnostics SL, Valencia, Spain). Optical densities (OD) were recorded at 405 nm after 60 min of substrate incubation, and samples with OD double that of the healthy control plants were considered positive. Finally, during the second cycle of study, to detect PPV, RNA was extracted from the same leaves used in the DASI-ELISA test using the Rneasy Plant Mini Kit^®^ (Qiagen, Hilden, Germany), and RT-PCR analysis was carried out using specific primers for the coat protein: VP337 (5′ CTCTGTGTCCTCTTCTTGTG 3′) and VP338 (5′ CAATAAAGCCATTGTTGGATC 3′). The enzymes used were Avian myeloblastosis virus reverse transcriptase and GoTaq^®^ polymerase (Promega, Madison, WI, USA). The RT-PCR parameters were 42 °C for 45 min (cDNA synthesis) followed by a step at 94 °C for 2 min; 35 cycles at 94 °C for 30 s, 55 °C for 30 s, and 72 °C for 30 s; and a final extension step at 72 °C for 5 min. RT-PCR-amplified products were separated by electrophoresis on 1% agarose gels in 40 mM Tris-acetate and 1 mM EDTA, pH8.0, and stained with Gel Red^®^ (Biotium, Fremont, CA, USA).

### 2.4. Experimental Design

The following treatments were assayed in this study: control (GFc) and infected “GF305” peach showing strong sharka symptoms (GFi); control “GF305” (GFc+Ga) grafted with “Garrigues” almond; and infected “GF305” grafted with “Garrigues” almond (GFi+Ga). In addition, “Garrigues” control (Gac) and PPV-inoculted samples (Gai) were also evaluated (Figure 1). Leaf samples from evaluation cycle 2 (Table 1) (a pool of leaves per replicate) were frozen in liquid nitrogen and stored at −80 °C.

### 2.5. Phytohormone Analysis

During the second cycle of study, after two months in the greenhouse but before senescence, leaf samples were frozen (−20 °C) for phytohormonal analysis. Three biological replications and three technical replications were assayed per treatment. The main classes of plant hormones, including three phytophormones related to growth (the cytokinin trans-zeatin (tZ) and the gibberellins GA_3_ and GA_4_) and four phytphormones related to stress (the ethylene precursor 1-aminocyclopropane-1-carboxylic acid (ACC), abscisic acid (ABA), salicylic acid (SA), and jasmonic acid (JA), were analyzed according to the protocol described by Albacete et al. [28,29] with some modifications. Briefly, 0.1 g of fresh plant material was homogenized in liquid nitrogen and dropped into 0.5 mL of a cold (−20 °C) extraction mixture of methanol/water (80/20, *v*/*v*). Solids were separated by centrifugation (20,000× *g*, 15 min) and re-extracted for 30 min at 4 °C in an additional 0.5 mL of the same extraction solution. Pooled supernatants were passed through a Sep-Pak Plus C18 cartridge (SepPak Plus, Waters, Milford, MA, USA) to remove interfering lipids and a part of the plant pigments and then evaporated at 40 °C under vacuum, either to near dryness or until the organic solvent was removed. The residue was dissolved in 1 mL of a methanol/water (20/80, *v*/*v*) solution using an ultrasonic bath. The dissolved samples were then filtered through 13 mm diameter Millex filters with a 0.22 µm pore size nylon membrane (Millipore, Bedford, MA, USA). A quantity of 10 µL of filtrated extract was injected into a U-HPLC-MS (Ultra-high performed liquid chromatography–tandem mass spectrometer) system consisting of an Accela Series U-HPLC (Thermo Fisher Scientific, Waltham, MA, USA) coupled to an Exactive mass spectrometer (Thermo Fisher Scientific, Waltham, MA, USA) using a heated electrospray ionization (HESI) interface. Mass spectra were obtained using the Xcalibur software version 2.2 (Thermo Fisher Scientific, Waltham, MA, USA). For quantification of the plant hormones, calibration curves were constructed for each analyzed component (1, 10, 50, and 100 µg L^−1^) and corrected for 10 µg L^−1^ deuterated internal standards. Recovery percentages ranged between 92 and 95%.

## 3. Results

### 3.1. Evaluation of Sharka Symptoms and PPV Detection

The results of Table 1 confirm the high susceptibility of “GF305” peach to PPV-D, since all inoculated plants showed clear symptoms and were ELISA-DASI and RT-PCR positive in sample GFi (Figure 1). Importantly, the resistance of the cultivar “Garrigues” to the PPV isolate assayed was confirmed by the absence of disease symptoms and negative ELISA-DASI and RT-PCR results in all of the Gac and Gai samples.

Furthermore, the results of this trial confirm that “Garrigues” induces a decrease in and subsequent elimination of symptoms in “GF305” peach, as can be seen in comparing the GFi+Ga sample with the GFi sample. Already in the first evaluation cycle, only two replications showed sharka symptoms, and after two cycles of study, only one did. However, ELISA and RT-PCR detected the presence of PPV-D in four plants during cycle 2 (sample GFi+Ga).

### 3.2. Growth-Related Phytohormones

**Figure 2:**
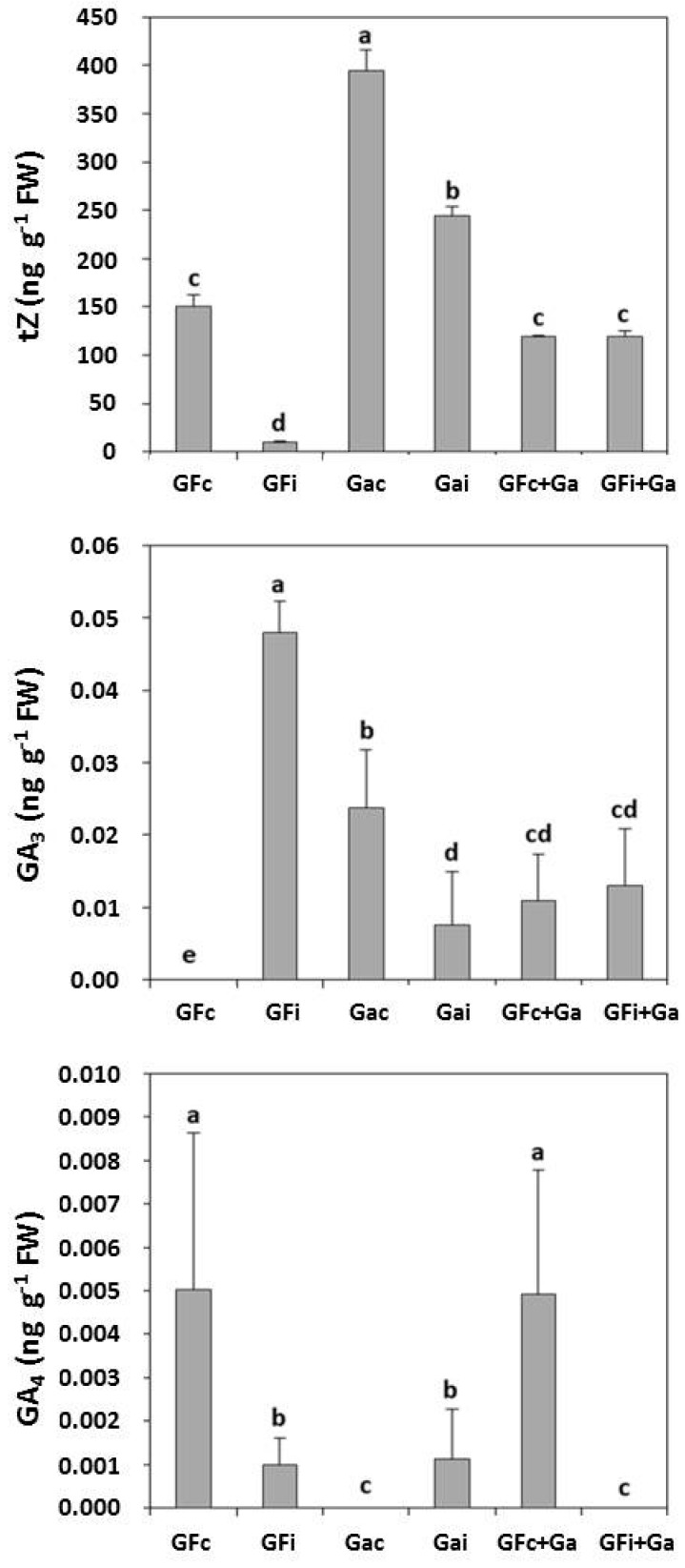
Concentrations (ng·g^−1^ fresh weight, FW) of the plant growth-related hormones, including the cytokinin trans-zeatin (tZ) and the gibberellins GA_3_ and GA_4_ in control (GFc) and infected (GFi) “GF305” peach showing strong sharka symptoms; control (Gac) and infected (Gai) “Garrigues” almond; control “GF305” grafted onto “Garrigues” almond (GFc+Ga); and infected “GF305” grafted onto “Garrigues” almond (GFi+Ga).

Figure 2 shows the concentrations of the phytohormones related to growth that were evaluated, including the cytokinin trans-zeatin (tZ) and the gibberellins GA_3_ and GA_4_. The assayed leaf samples were collected during the second cycle of study after two months in the greenhouse before senescence.

Despite the low concentrations of the gibberellins analyzed, a sharp increase in GA_3_ was observed in “GF305” after PPV-D inoculation. However, results showed only a small increase in GA_3_ in the infected “GF305” grafted onto “Garrigues”. Additionally, the concentrations of GA_3_ were lower in both control and inoculated “Garrigues”. In general, grafting “Garrigues” increased the GA_3_ levels. Interestingly, a decrease in GA_4_ was observed after the PPV-D inoculation of “GF305” peach in both treatments (control and “G305” peach grafted with “Garrigues”). The GA_4_ levels were more stable in control and inoculated “Garrigues”, with a reduced effect observed upon grafting “Garrigues” onto “GF305”.

Regarding the cytokinins, PPV-D inoculation produced an important decrease in the concentration of tZ in the “GF305” plants, although this decrease was not observed after “Garrigues” grafting. In contrast, the presence of “Garrigues” kept the tZ concentrations in “GF305” similar to the levels in the healthy control. The tZ concentrations were more stable in both the control and inoculated “Garrigues” plants. It can be also noted that in almond, independent of the virus, the tZ concentration is higher.

### 3.3. Stress-Related Phytohormones

**Figure 3:**
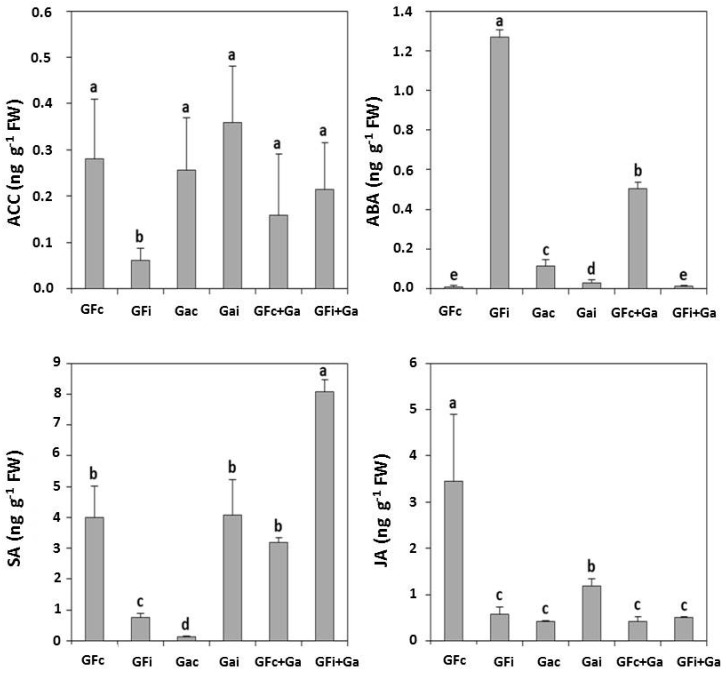
Concentrations (ng·g^−1^ fresh weight, FW) of stress-related hormones, including the ethylene precursor 1-aminocyclopropane-1-carboxylic acid (ACC), abscisic acid (ABA), salicylic acid (SA), and jasmonic acid (JA) in control (GFc) and infected (GFi) “GF305” peach showing strong sharka symptoms; control (Gac) and infected (Gai) “Garrigues” almond; and control “GF305” grafted onto “Garrigues” almond (GFc+Ga) and infected “GF305” grafted with “Garrigues” (GFi+Ga).

Figure 3 shows the results of the analysis of plant hormones related to stress, including the ethylene acid precursor 1-aminocyclopropane-1-carboxylic acid (ACC), abscisic acid (ABA), salicylic acid (SA), and jasmonic acid (JA).

These four phytohormones showed similar trends in the treatments assayed. PPV-D inoculation produced an important decrease in the ACC, SA, and JA concentrations in “GF305” that was not observed after “Garrigues” grafting. In addition, importantly, PPV inoculation produced an increase in the concentrations of ABA in “GF305”. In contrast, “Garrigues” grafting produced an increase in ACC, SA, and JA in “GF305” after PPV inoculation. The concentrations of these hormones also increased after the PPV-D inoculation of “Garrigues” almond. However, in the case of SA, the phytohormone differences were higher in comparison with JA, while the differences in ACC were not statistically significant.

## 4. Discussion

Sharka evaluation results confirmed previous findings from Rubio et al. [8] indicating that grafting the almond cultivar “Garrigues” onto “GF305” peach seedlings heavily infected with PPV can progressively reduce disease symptoms and virus accumulation. This response appears to be induced after “Garrigues” grafting [8]. The response consists of a decrease in symptoms and an absence of other reactions producing necrosis or a hypersensitive response (HR) inside the identified as Systemic Acquired Resistance (SAR) [30]. In addition to SAR, Induced Systemic Resistance (ISR) is the other broad-spectrum systemic resistance described in plants induced by pathogens [31]. However, the response observed in our studies was different from SAR and ISR, which are locally and systemically activated after virus infection and produce necrosis, which was not observed in our case.

In the case of *Prunus*/PPV interaction, only one case of HR-type resistance has been described in European plum (*P. domestica* L.), in the “Jojo” genotype [32] hybrid “K-4” [33]. These authors describe the necrosis of plum plants infected with PPV-M isolates. This type of resistance is being used in breeding programs to obtain new resistant European plum varieties [34]. The other type of resistance characterized in *Prunus* is in apricot (*P. armeniaca* L.), and it is associated with RTM genes (Restricted *Tobacco etch virus* (TEV) movement) within the MATH (*Methionine adenosyltransferases*) domain family [35,36,37]. This last response is more similar to that observed in our study than the HR identified in European plum.

After the phytohormone analysis, our results showed the significant involvement of SA in the induced resistance response in peach after “Garrigues” grafting. An SA increase was clearly induced when there was a clear decrease in symptoms in PPV-D-inoculated “GF305” peach grafted with “Garrigues” (Figure 1). This response was similar in the case of “Garrigues” almond after PPV-D inoculation in the absence of almond leaf symptoms (Figure 1). On the other hand, the PPV-D inoculation of “GF305” peach resulted in a drastic decrease in SA along with the presence of PPV symptoms (Figure 1). Finally, “Garrigues” grafting produced a slight increase in SA in control peach (Figure 1).

The link between SA responses and systemic resistance responses (SAR and ISR) has been described in different plant species [30,31]. SA participates in the establishment of local and systemic resistance [38]. Furthermore, SA also participates in the regulation of small interfering RNAs (siRNAs) and favors the accumulation of reactive oxygen species (ROS), the synthesis of PR proteins, callose deposits, and the hypersensitive response related to the apoptosis of infected cells or those close to the infection zone [39]. Gómez-Muñoz et al. [40], for instance, described that the resistance of sour orange to *Citrus tristeza virus* is mediated by both the salicylic acid and the RNA silencing defense pathways. All of these processes lead to the aforementioned SAR. In addition, mutations in the pathway of synthesis or SA signaling generate plants susceptible to viral infections even with the presence of *R* genes [41]. Moreover, overexpression of SA synthesis genes or their exogenous application improves basal immunity by delaying the establishment of viral infection [42].

An alternative form of SA-induced resistance different from SAR is called extreme resistance (ER), which is characterized by the elimination of the virus and the absence of necrotic lesions in plants that possess *R* genes. This type of resistance has been described in tomato plants infected with TBSV (*Tomato bushy stunt virus*) in which the so-called P19 protein forms a complex with a siRNA to trigger ER by silencing the viral suppressor RNA of TBSV [43]. This mechanism of resistance mediated by SA seems to fit more closely with the phenotypic and hormonal data observed in this study. On the other hand, transgenic lines that overexpress viral suppressor proteins P1/HC-Pro have also been shown to reduce the SA-mediated response and PPV-derived siRNA levels [44]. However, the proteins involved in the production of small-sized RNA (dicer-like proteins, DCL) appear to be independent of the resistance mediated by SA in *Arabidopsis*, where DCL mutants have been described that reduce the viral titer in treatments with SA [41]. This seems to indicate that SA triggers several defense mechanisms independent of DCL.

An SA-induced response based on the restriction of PPV movement is compatible with the results observed in this study, although it is different from SAR and ISR. The accumulation of SA also activates a series of regulatory proteins such as NPR1/NIM and transcription factors that control the expression of defense genes such as the genes of PR (Pathogen-Related) proteins [30,31]. The repression of viral replication produced by SA should be mediated by siRNA, as has been described in different plant species and viruses [45]. Recently, Cueto-Ginzo et al. [46] indicated that exogenous SA treatment delays initial infection and alterations induced by *Maize dwarf mosaic virus* in maize. In contrast, Ma and Ma [47] described the manipulation of SA levels to suppress plant defense mechanisms in plants.

Regarding the effect of grafting, Heil and Ton [48] described the long-range movement of SAR-type resistance within the plant. In a similar vein, Guan et al. [49] have also indicated the possibility of transmitting SAR-type resistance via xylem from the rootstock to the variety by modifying the levels of antioxidant enzymes and the presence of molecules such as SA. In our case, the SA-induced response was likely transmitted via phloem.

Our results also highlight the role of the growth-related cytokinin trans-zeatin in the induced resistance process. Grafting “Garrigues” onto “GF305” maintained the level of tZ after PPV inoculation, whereas inoculation typically produced a drastic decrease in tZ in “GF305” peach. The action of cytokinins at the physiological level serves to induce cell proliferation and delay leaf senescence. At the immunological level, cytokinins play an important role in resistance against biotrophs, acting together with the SA in its signaling pathway. The anti-biotroph effect of cytokinins has been described as being dependent upon SA and even dose-dependent [50]. Accordingly, different authors have described the role of cytokinins in resistance to *Pseudomonas syringae* in *Arabidopsis* [51] and tobacco [52]. To the contrary, Ma and Ma [47] have described that the modulation of the cytokinin pathway suppresses defense mechanisms in plants. Cytokinins have been identified as key regulators in plant–microbe–insect interactions in terms of plant growth and defense [53]. In our case, the SA signaling should activate the cytokinin response to induce PPV resistance.

JA also participates in the response against viruses. Blocking the synthesis of JA has been found to favor the accumulation of Potato virus Y in the initial stages of potato infection [54]. The application of JA at the beginning of PVY–PVX (*Potato virus Y*–*Potato virus X*) double infection also favors resistance against these viruses, although its application in non-initial stages favors susceptibility [55].

In addition, in studies in *Nicotiana benthamiana*, where pretreatment with JA was followed by SA treatment, an increase in resistance against *Tobacco mosaic virus (*TMV) was observed [56]. However, Oka et al. [57] have suggested that JA signaling is not directly responsible for resistance to TMV in tobacco. Instead, these researchers argue that this signaling is indirectly responsible for viral resistance through the partial inhibition of SA-mediated resistance conferred by the *N* gene, and that a balance between endogenous JA and SA levels is important for determining the degree of resistance to the virus. These findings corroborate our results regarding the increase in JA concentration in induced PPV resistance in peach. Additionally, Ma and Ma [47] have described the manipulation of JA signaling to promote pathogen dissemination in susceptible genotypes.

The response observed in the analysis of the ethylene precursor ACC was not as clear as the SA and JA responses. Ethylene has been found to play a dual role action in senescence and the plant response against necrotrophs [58]. Ethylene does not seem essential for the establishment of plant resistance against viruses, and several articles show that ethylene is responsible for symptom development, as seen in cucumber infected with CMV (*Cucumber mosaic virus*) [59]. Ma and Ma [47] also described the manipulation of ethylene levels to promote pathogen infection. Studies in *N. tabacum* have detected the presence of the ethylene precursor ACC accumulated locally near necrotic areas, indicating its contribution to the formation of the lesion [60]. This response was not observed in our *Prunus* analysis.

The responses observed in the rest of the growth-related phytohormones evaluated (GA_3_ and GA_4_) were not as clear as the tZ response. However, a certain effect was also observed. Gibberellins participate in germination, stem elongation, and flowering. Their role in the plant immune response seems to depend on the balance between SA and JA/ethylene [16]. Ent-kaurene oxidase is involved in the biosynthesis of gibberellins [61], and this enzyme interacts with RDV viral capsid proteins (*Rice dwarf virus*), thereby reducing GA_1_ levels and phytoalexin biosynthesis, promoting viral replication [62], as we observed in our analysis of GA_3_.

Finally, concentrations of ABA in advanced stages of viral infection can induce the suppression of the SA and JA signaling pathways. As a result, if ABA levels decrease, this favors the immune action mediated by these hormones. We can state that ABA plays a negative role in the response to infection in plants with resistance genes against viruses [63]. The decrease in ABA observed after “Garrigues" grafting favored the oxidative conditions inside the cell, which, in turn, induced the monomeric form of NPR1; this form is capable of being translocated to the nucleus, indicating the signaling of the responses mediated by SA [64]. Ma and Ma [47] also described the manipulation of ABA levels by the pathogen to promote infection.

These results confirm the new opportunities for crop improvement and food security using grafting as indicated by Albacete et al. [65]. The induced resistance observed is a more suitable strategy for PPV-D control than the previously described tolerance [6] or the introduction of PPV resistance via genetic transformation [5]. New studies are necessary, however, to validate these results against PPV-D isolates in the more aggressive M isolates [3,5].

## 5. Conclusions

The study of the hormonal balance in the control “GF305” peach and the “GF305” peach inoculated with a PPV-D isolate, both grafted and not grafted with the almond cultivar “Garrigues”, showed the important differences between treatments. PPV inoculation produced a significant increase in GA_3_ and ABA and a decrease in the other phytohormones analyzed, including tZ, GA_4_, ACC, SA, and JA. These imbalances were related to the virus infection and the presence of chlorosis symptoms, particularly in the case of the ABA concentration. Additionally, grafting “Garrigues” onto “GF305” produced an increase in GA_3_, GA_4_, SA, and ABA and a decrease in the rest of the phytohormones analyzed—tZ, ACC, and JA. However, grafting “Garrigues” almond onto the PPV-inoculated peach “GF305” produced the opposite effect in some phytohormones, resulting in an increase in tZ, SA, and JA. Our results show the significant involvement of SA in the induced resistance response in peach after “Garrigues” grafting. A significant decrease in ABA and increase in ACC was also observed in relationship with the elimination of symptoms. This trend was also detected in the case of the inoculation of “Garrigues”. Our results also show the implication of tZ and JA in virus infection and the induced resistance in peach from almond through SA-induced resistance. This SA-induced resistance, consisting of a decrease in symptoms and an absence of other reactions producing necrosis, seems to be different from SAR and ISR. SA signaling should activate a cytokinin response to induce PPV resistance linking plant growth and defense. JA also participates in the response against PPV. Finally, we also observed an indirect effect on ABA and ACC concentrations and an unclear effect on gibberellins. Further studies are necessary, however, to validate these results against PPV-D isolates in the more aggressive PPV-M isolates.

## Figures and Tables

**Table 1:** Behavior against PPV in the plant material assayed. The samples assayed included control (GFc) and infected (GFi) “GF305” peach showing strong sharka symptoms; control (Gac) and infected (Gai) “Garrigues” almond; and control “GF305” grafted onto “Garrigues” almond (GFc+Ga) and infected “GF305” grafted onto “Garrigues” almond (GFi+Ga). The plant material evaluated appears in bold type.

		Cycle 1		Cycle 2		
Treatments	Samples	Symptoms ^1^	ELISA ^2^	Symptoms ^1^	ELISA ^2^	RT-PCR ^3^
**GF305** Control	GFc	0 (0.0)	0 (0.052)	0 (0.0)	0 (0.067)	0
**GF305** + PPV	GFi	8 (3.3)	8 (3.529)	8 (4.2)	8 (3.187)	8
**GF305** Control < **Garrigues**	Gac	0 (0.0)	0 (0.061)	0 (0.0)	0 (0.059)	0
**GF305** + PPV < **Garrigues**	Gai	0 (0.0)	0 (0.052)	0 (0.0)	0 (0.052)	0
**GF305** Control < Garrigues	GFc+Ga	0 (0.0)	0 (0.059)	0 (0.0)	0 (0.058)	0
**GF305** + PPV < Garrigues	GFi+Ga	2 (1.0)	2 (1.222)	1 (1.0)	4 (1.487)	4

^1^ Number of replicates with sharka symptoms, with the mean values of the repetitions with symptoms on a scale of 0 to 5 between parentheses; ^2^ Number of replicates that were ELISA-DASI positive, with the mean OD values of the assayed repetitions between parentheses; ^3^ Number of RT-PCR positive replicates.
